# Supplementary material for: MetaRibo-Seq measures translation in microbiomes
Source: Nat Commun. 2020 Jun 29;11:3268. doi: 10.1038/s41467-020-17081-z (PMC7324362; doi:10.1038/s41467-020-17081-z)
Supplement: Supplementary file 10 — Supplementary Data 7 [file 41467_2020_17081_MOESM10_ESM.zip › File2/Confidence_VeryHigh_Taxonomy/35277_out.krona.html]

Javascript must be enabled to view this page.

members
magnitude
magnitudeUnassigned
count
unassigned
taxon
rank

35277\_out

6

superkingdom
6
2

976
phylum
6

class
6
200643

171549
order
6

family
6
171552

838
6
genus


SRS012969\_contig\_number\_contig-100\_166.228772SRS015794\_contig\_number\_contig-100\_226.90060SRS023526\_contig\_number\_13504SRS049959\_contig\_number\_37551SRS049959\_contig\_number\_47030SRS077849\_contig\_number\_contig-100\_168.185937
species
6
2292365
